# Cyclodextrins Allow the Combination of Incompatible Vancomycin and Ceftazidime into an Ophthalmic Formulation for the Treatment of Bacterial Keratitis

**DOI:** 10.3390/ijms221910538

**Published:** 2021-09-29

**Authors:** Yassine Bouattour, Florent Neflot-Bissuel, Mounir Traïkia, Anne-Sophie Biesse-Martin, Robin Frederic, Mouloud Yessaad, Mireille Jouannet, Mathieu Wasiak, Philip Chennell, Valerie Sautou

**Affiliations:** 1 Université Clermont Auvergne, CHU Clermont Ferrand, Clermont Auvergne INP, CNRS, ICCF, F-63000 Clermont-Ferrand, France; ybouattour@chu-clermontferrand.fr (Y.B.); vsautou@chu-clermontferrand.fr (V.S.); 2 CHU Clermont-Ferrand, Pôle Pharmacie, F-63000 Clermont-Ferrand, France; bissuelflorent@orange.fr (F.N.-B.); myessaad@chu-clermontferrand.fr (M.Y.); mjouannet@chu-clermontferrand.fr (M.J.); mwasiak@chu-clermontferrand.fr (M.W.); 3 Université Clermont Auvergne, CNRS, SIGMA-Clermont, ICCF, F-63000 Clermont-Ferrand, France; mounir.traikia@uca.fr (M.T.); a-sophie.biesse-martin@uca.fr (A.-S.B.-M.); 4 Université Clermont Auvergne, Inserm U1071, INRA USC2018, F-63000 Clermont-Ferrand, France; frobin@chu-clermontferrand.fr

**Keywords:** cyclodextrins, vancomycin, ceftazidime, ophthalmic solution, bacterial keratitis, nuclear magnetic resonance, design of experiments

## Abstract

Ceftazidime (CZ) and vancomycin (VA) are two antibiotics used to treat bacterial keratitis. Due to their physical incompatibility (formation of a precipitate), it is not currently possible to associate both molecules in a single container for ophthalmic administration. We firstly characterized the incompatibility then investigated if 2-hydroxypropyl-beta (HPβCD) and 2-hydroxypropyl-gamma cyclodextrins (HPγCD) could prevent this incompatibility. The impact of pH on the precipitation phenomena was investigated by analysing the supernatant solution of the mixture using high performance liquid chromatography. A characterization of the inclusion of CZ with HPγCD using ^1^H nuclear magnetic resonance (NMR), and VA with HPβCD using ^1^H-NMR and a solubility diagram was performed. A design of experiment was built to determine the optimal conditions to obtain a formulation that had the lowest turbidity and particle count. Our results showed that VA and CZ form an equimolar precipitate below pH 7.3. The best formulation obtained underwent an in-vitro evaluation of its antibacterial activity. The impact of HPCDs on incompatibility has been demonstrated through the inclusion of antibiotics and especially VA. The formulation has been shown to be able to inhibit the incompatibility for pH higher than 7.3 and to possess unaltered antibacterial activity.

## 1. Introduction

**Figure 1:**
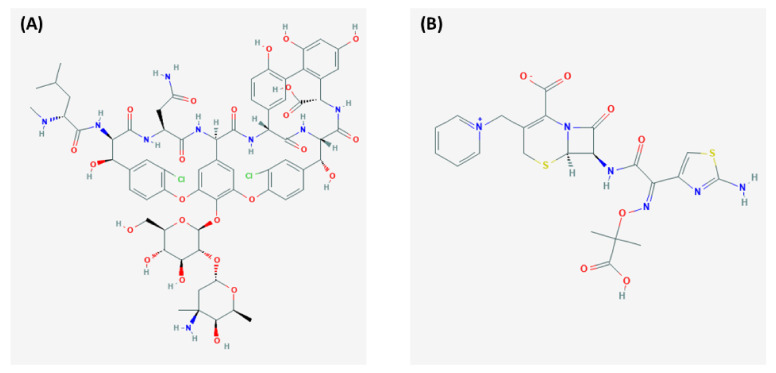
Chemical structure of vancomycin (**A**) and ceftazidime (**B**). Publicly available from [14].

Infectious keratitis is a severe sight-threatening ocular infection with increasing prevalence worldwide, ranging from 2.5 to 799 cases per 100,000 population/year [1], despite it also probably being under reported. It is one of the most important causes of corneal opacifications, which is the second most common cause of blindness after cataracts, reportedly causing up to 5% of all blindness cases [2]. Of these infections, bacterial keratitis (BK) represents the most common (>90%) type of infectious keratitis in most regions of the world [1]. This infection is mainly caused by extended-contact lens wear and ocular trauma, even if other causes have been documented [3,4]. Symptoms include sudden pain in the eye, unusual eye redness, reduced vision, increased light sensitivity, excessive tearing and eye discharge, and in the end if without treatment, blindness. The most commonly isolated pathogens responsible for BK are still *Pseudomonas aeruginosa*, *Staphylococcus areus*, but other organisms are on the rise such as *coagulase-negative Staphylococci,* as well as *Streptococci spp.* and *Moraxella spp.* [1,2,5,6]. Unfortunately, identifying the causal pathogen to be able treat it with the appropriate antibiotic takes laboratory time that the patient often does not have, which is why an empiric treatment of antibiotic eye drops covering the spectra of the most common bacteria is recommended [7,8,9]. Those eye drops are given to the hospitalized patient every 5 to 10 min the first hour, then every hour (day and night) for 48 h before being reduced to hourly (daytime only) for several days. Amongst the active pharmaceutical ingredients (API) that can be used are vancomycin (VA) and ceftazidime (CZ), used at concentrations ranging usually from 25 to 50 mg mL^−1^. VA is a much-used glycopeptide that acts by blocking peptidoglycan synthesis of the bacterial wall, leading to leakage of intracellular components, which gives it bactericidal properties [10]. However, due to its high molecular weight, it cannot penetrate the pores of the outer membrane of Gram-negative bacteria. Its spectrum of action is therefore limited to Gram-positive bacteria such as *Staphylococcus aureus* or *Streptococcus spp.*. CZ is a third-generation cephalosporin (group of beta-lactam antimicrobials) that possess a beta-lactam ring that binds to the penicillin-binding protein and inhibit its normal activity. Unable to synthesize a cell wall, the bacteria die [11]. CZ is active against Gram-positive and Gram-negative bacteria, including those resistant to other antibiotics. Very importantly, CZ also is also effective against *Pseudomonas aeruginosa.* The chemical structures of these API are presented Figure 1. Both these drugs are only marketed for intravenous administration, meaning that compounding pharmacies must prepare the eye drops needed for treatment. Unfortunately, these two API cannot be given simultaneously as they are incompatible when mixed together, forming a cloudy white precipitate [12] that cannot be safely administered to the patient. This incompatibility also manifests itself between VA and other cephalosporins [12] as well as when the drugs are administered sequentially yet separately [13], meaning that in practice the nurse administering each eye drop of the different antibiotic must wait several minutes between the two antibiotics, which is a time consuming and inefficient task.

Cyclodextrins (CDs) are cyclic natural oligosaccharides consisting of 6 (αCD), 7 (βCD) or 8 (γCD) glucose monomers linked via α-1,4-glycosidic bonds. The molecules are shaped similar to a doughnut with a hydrophilic outer surface and a somewhat lipophilic central cavity. The natural CDs and their complexes being rather poorly water-soluble, derivatives such as for example 2-hydroxypropyl-βCD (HPβCD) and 2-hydroxypropyl-γCD (HPγCD) with solubilities higher than 500 mg/mL have been chemically prepared. Many uses have been described for the compounds, including as a useful excipient for drug preparation and delivery, as they can temporarily camouflage undesirable API physiochemical properties such as low aqueous solubility or poor stability through formation of drug/CD inclusion complexes, for example for parenteral medications [15]. Their use as also been described for drug delivery of ocular therapeutics, for surface, anterior and posterior segments of the eye [16,17,18,19] and are considered more and more as safe and effective for this use. Interestingly, their use as potential drug carriers that could block the precipitation interaction between VA and CZ has to our knowledge not been published. As VA has been shown to complex itself with βCD [20,21], and CZ with γCD [22], the rationale of this study was to use both β and γCD (in their 2-hydroxypropyl derivative) to investigate their potential for blocking the problematic incompatibility between VA and CZ, and thus pave the way for the development of a novel ophthalmic solution combining these two useful antibiotics.

## 2. Results

### 2.1. Preliminary Investigation of Ceftazidime-Vancomycin Incompatibility

For 25 mg mL^−1^ CZ solutions, a variation of the pH between 4 and 9 did not cause any visible precipitation. However, for 25 mg mL^−1^ VA solutions, a highly visible precipitation was noticed for pH values between 7.3 and 8.4. When combined, the CZ/VA mixture (target concentration of 25 mg mL^−1^) was completely incompatible (visible white precipitation) for pH ranging from 3 to 8.4, but we noticed that the precipitation was less intense when the pH was between 7.3 and 8.4. The mixture showed no signs of visible precipitation at a pH above 8.4 (See Supplementary Materials File S1).

A quantification of both antibiotics in the supernatant liquid after centrifugation, performed for a final pH of the mixture at pH = 7 and pH = 8, showed reduced concentrations of both VA and CZ, but especially for VA for which concentrations were reduced by 27.7 and 50.0% at pH = 7 and pH = 8, respectively (see Table 1). When the concentrations are expressed in mmol L^−1^, it can be seen that at pH 7 VA and CZ lost an equal amount of matter (12.05 and 11.51 mmol L^−1^) whereas at pH 8 the loss of VA was 4 times greater than CZ (8.33 versus 2.31 mmol L^−1^). The analysis of some recovered precipitate showed the presence of both VA and CZ. The amount of each compound in the tested precipitate was found to be of 0.03 µmoL.

### 2.2. Antibiotics Inclusion Characterization in Hpcds

#### 2.2.1. Nuclear Magnetic Resonance (NMR) Analyses

##### ^1^H NMR-Spectroscopy Chemical Shifts Measurements

**Figure 2:**
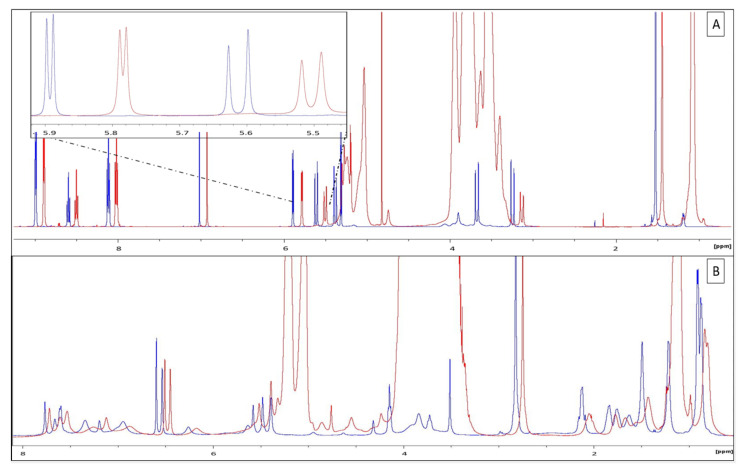
Nuclear magnetic resonance spectra of (**A**) ceftazidime (blue curve) and ceftazidime/hydroxypropyl-γ-cyclodextrins (red curve) at 50 mg/mL at pH 4, (**B**) vancomycin (blue curve) and vancomycin/hydroxypropyl-β-cyclodextrins (red curve) at 50 mg/mL at pH 4.

By superimposing the spectra of a 50 mg/mL CZ and the CZ/HPγCD (1:3 ratio) solution at pH 4, variations between the two spectra, as a shift in the peaks of CZ towards the lowest frequencies (high field) was clearly present. This was observed even more so on the enlargement between 5.5 and 6 ppm. The RMN analysis revealed a decrease in the resolution and widening of the peaks. Those shifts were also found at pH 8 for CZ confirming its inclusion in HPγCD. Similar results were also found when comparing VA and the VA/HPβCD (1:5 ratio) solutions at 50 mg mL^−1^ and at pH 3 (Figure 2). Full chemical shift details and peak attribution are supplied in Supplementary Materials S1 for CZ and CZ/HPγCD. As VA is a larger and more complex molecule (1449.2 g/mol^−1^), signal analysis is much more difficult, especially as the peaks also broaden after the inclusion, and chemical shift analysis was not performed. In addition, the average molecular weight of the cyclodextrins were estimated by NMR and were found to be of 1527–1568 g/mol^−1^ (HPßCD) and 1690–1705 g/mol^−1^ (HPγCD).

##### ^1^H NMR-Diffusion Measurements (^1^H-DOSY)

**Figure 3:**
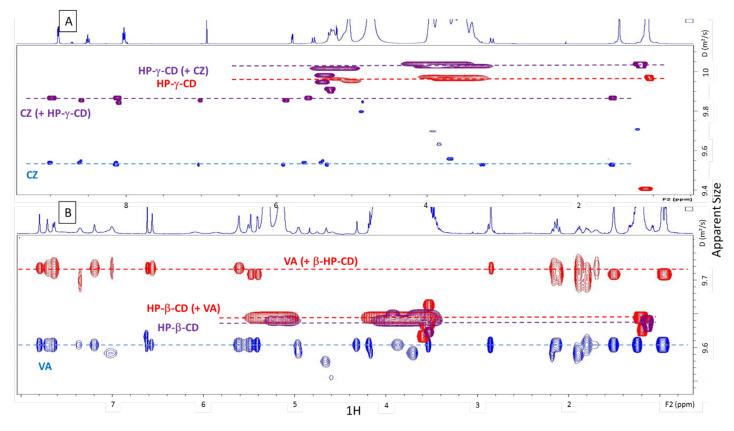
The ^1^H-diffusion ordered spectroscopy spectra of (**A**) ceftazidime (CZ, blue curve), hydroxypropyl-γ-cyclodextrins (HPγCD, red curve) alone and in mixture at 50 mg ml^−1^ and at pH 4 (purple curve) and (**B**) of vancomycin (VA, blue curve), hydroxypropyl-β-cyclodextrins (HPβCD, purple curve) alone and in mixture at 50 mg/mL and at pH 3 (red curve).

The DOSY superimposition of the spectra of CZ, HPγCD and the CZ/HPγCD mixture showed an increase in the apparent size of CZ and HPγCD in the mixture compared to their initial size when analysed alone. The same results were found when analysing VA, HPβCD and the VA/HPβCD mixture solution. The apparent size of both antibiotics in their respective cyclodextrins solution was greater than the apparent size of CDs in the mixture (Figure 3).

#### 2.2.2. Influence of HPβCD/Vancomycin Molar Ratio on Vancomycin Inclusion and Precipitation

**Figure 4:**
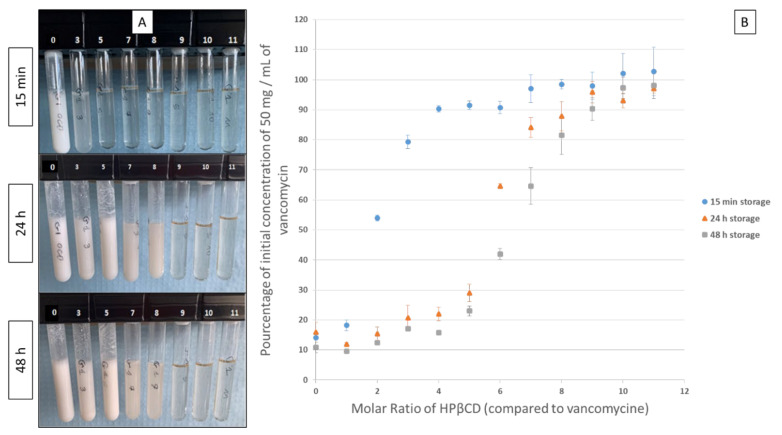
Influence of hydroxypropyl-β-cyclodextrins (HPβCD)/vancomycin molar ratio on vancomycin inclusion and precipitation at pH 8 as function of the HPβCD/vancomycin molar ratio immediately after preparation and after up to 48 h hours of storage: (**A**) visual aspect (the white numbers indicate the HPβCD/vancomycin molar ratio of the solutions and (**B**) vancomycin concentration (*n* = 3, mean ± standard deviation). h = hour.

In order to investigate the impact of HPβCD concentrations on VA solubility at pH 8 (at a concentration of 50 mg/mL), various molar ratios of HPβCD/VA were tested for visible signs of precipitation after up to 48 h of storage at 22 °C. The results presented in (Figure 4A) showed that after 15 min, a molar ratio of 7:1 can solubilize the molecule (97.0% of theoretical concentration) and avoid visible signs of precipitation. However, this ratio did not prevent the solution from precipitating after 24 h hours of storage. Indeed, a molar ratio of 10:1 was found to be necessary to maintain solution limpidity even after 48h of storage, which was confirmed by the vancomycin quantification results (97.3% of theoretical concentration in the supernatant), thus indicating complete vancomycin solubility, see (Figure 4B).

### 2.3. Determination of the Optimum Preparation Method Using Design of Experiments

The mean impact of the parameters tested during the 84 experiments on visual aspect, turbidity, particulate count and osmolality was calculated when these parameters varied from the minimum to the maximum value, as represented in Table 2. Full experimental data are supplied in the Supplementary Materials File S1. The two factors that had the most positive impact (decreasing physical incompatibility signs) were the pH of the final mixture and the concentration of the cyclodextrins, and the factor which had the most negative impact (increasing incompatibility signs) was the pH of the CD-antibiotic complexation. The stirring time had no interpretable effect.

**Figure 5:**
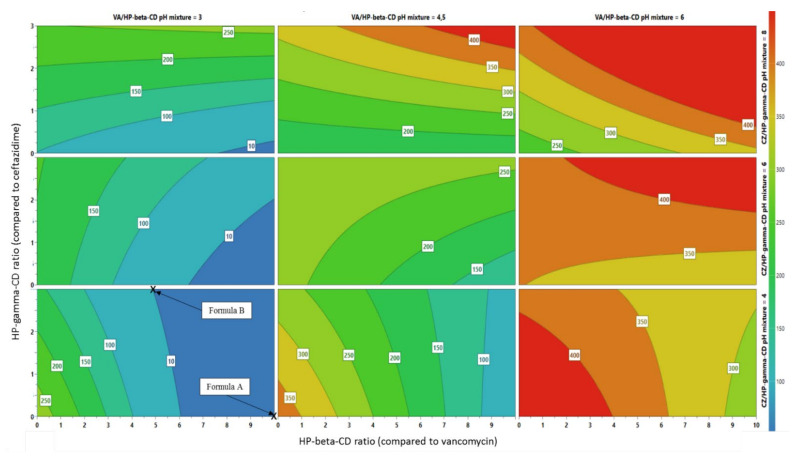
Variation of turbidity as function of simultaneous variation of parameters when stirring time of hydroxypropyl-β-cyclodextrins (HPβCD)—vancomycin (VA) fixed at 2 h, stirring time of Hydroxypropyl-γ-cyclodextrins (HPγCD–ceftazidime (CZ) fixed at 0.5 h and final pH of the solution is at 8.

In order to visually comprehend the impact of these parameters and determine the optimum preparation method, a four-dimensional representation was elaborated, using turbidity as the main response factor (see Figure 5).

The optimum conditions yielding the lowest turbidity values are visualized in blue, and the highest (worst) in red. We found that the best conditions were when stirring time of VA/HPβCD was set at 2 h, stirring time of CZ/HPγCD was at 30 min, initial pH mixture of VA/HPβCD at 3 and initial pH mixture of CZ/HPγCD at 4. We fixed the final pH solution at 8 to be in the ocular-tolerated pH range, reported to be between 5 and 8.5 [23,24]. As such, two optimal methods were selected:

Formula A: VA 25 mg mL^−1^, CZ 25 mg mL^−1^, 50 mM phosphate buffer at pH 8, HPβCD at 250 mg mL^−1^ (corresponding to a ratio of 10:1 compared to vancomycin);

Formula B: VA 25 mg mL^−1^, CZ 25 mg mL^−1^, 50 mM phosphate buffer at pH 8, HPβCD at 125 mgmL^−1^ (corresponding to a ratio of 5:1 compared to vancomycin) and HPγCD at 216.8 mgmL^−1^ (corresponding to a ratio of 3:1 compared to ceftazidime).

### 2.4. Validation of the Preparation Method

The two formulas selected according to the DOE results were prepared in bigger volumes and subjected to pH variations and refrigerated storage. The variation of pH from 7.5 to 9 showed no precipitation for the two formulas. However, decreasing pH below 7.5 resulted in precipitation of the prepared solution for the two formulas. In addition, if placed at 5 °C, we found a precipitate in Formula A but none for Formula B, demonstrating that the latter is the better candidate. After sterilization of Formula B by filtration through a 0.22 µm filter, the loss of concentration was found to be negligible (1.48% for CZ and 0.38% for VA).

### 2.5. Verification of the Efficiency Mixture of the Optimized Formula

**Figure 6:**
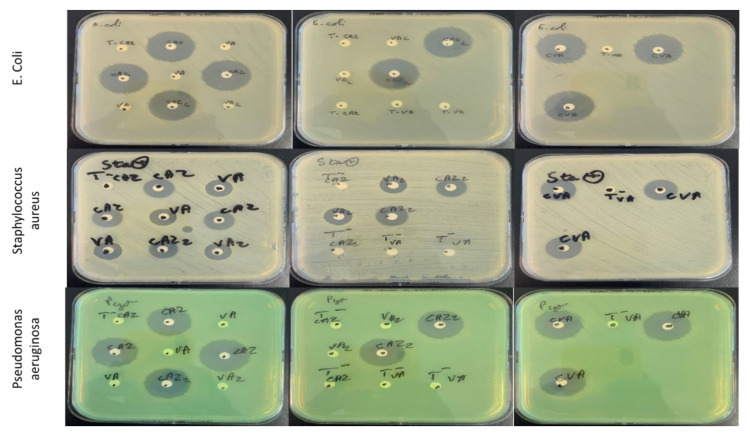
Culture of *E. coli* ATCC 25922, *S. aureus* ATCC29213 and *P. aeruginosa* ATCC27853 after 18 h of incubation at 35 °C with different antibiotic solutions: T-VA: negative control pH 3 and T-CVZ: pH 4; CZ: Ceftazidime; VA: Vancomycin; CZA2: Ceftazidime + HPβCD at pH 4; VA2: Vancomycin + HP γCD at pH 3; CVA: Formula B.

In order to investigate the antimicrobial efficacy of the final formulation, disk diffusion tests were performed on three bacterial strains. The results are presented in Table 3 and illustrated in Figure 6. Formula B presented the same results compared to reference solution of CZ and VA solution, and showed a preserved activity against *Escherichia coli* ATCC 25922), *Staphylococcus aureus* ATCC 29213 and *Pseudomonas aeruginosa* ATTC 27853 cultures.

## 3. Discussion

In this study, we aimed to develop a formulation enabling a physically compatible mix of CZ and VA, each at a concentration of 25 mg mL^−1^ by using HPCD, in order to obtain an ophthalmic solution that can be used treat BK. This was achieved as we obtained a solution mix of CZ and VA that was physically devoid of any particulate matter for pH higher than 7.3 compared to pH 8.4 without HPCD. However, for pH lower than 7.3, even the use of HPγCD and HPβCD at high concentrations did not prevent the incompatibility.

Incompatibility of CZ and VA has been largely reported since the 90’s, especially for intravitreal injection [13,25,26,27] as well as for parenteral [28] and peritoneal dialysis solutions [29], but its exact mechanism is still not well known. It has been hypothesized that VA precipitation could be due to the presence of sodium carbonate in the CZ formulation used to alkalinize the solution, but was also reported when the solution is carbonate free [12,13]. It was also found to be temperature dependent occurring at 37 °C but less at ambient temperature, and solvent dependent occurring more with balanced salt solution (BSS) than normal saline solution [30]. VA precipitation has also been reported in association with many other drugs such as cephalosporins, gelatine fluid, in BSS or in a pH of 7.5 [31,32,33]. In this study, we first investigated the incompatibility of the two molecules, using pharmaceutical sources containing no excipients (VA) or only sodium carbonate (CZ). The preliminary observations corroborated the theoretical data namely that: CZ is soluble at 50 mg mL^−1^ at whatever pH, the VA however precipitates between pH 7.3 and 8.4, the CZ/VA mixture precipitates from pH 4 to pH 8.4. After pH 8.4, the solution becomes clear. By analysing computed ionization data for each molecule [34], we noticed that the two antibiotics are of net opposite charges for a pH between 4 and 7.3 (−1 for CZ and +1 for VA). Indeed, as described by Johnson and Yalkowsky [35] the net charge of VA will be at +1 when pH between 4 and 7 and began to decrease after pH 7 to reach 0 at pH 8.3. In fact, VA will have two positive chemical moieties (amine functional group) and one negative chemical moiety (acid functional group), which is the total opposite of the ceftazidime at the same range of pH [34]. They would combine over this pH range forming a precipitate. Between 7.3 and 8.4, CZ remains negative while the main part of VA becomes molecular, causing a huge drop in its solubility. It would then be this molecular part which would precipitate predominantly over this pH range, and would therefore shift the HPCD complexation equilibrium as the free species precipitates. After pH 8.4, the average charge of VA becomes negative similar to that of CZ, which explains why they would no longer associate and therefore no longer precipitate either by incompatibility or by low solubility, as the ionized form is more water soluble. In order to validate the theory, a quantification of each antibiotic in the supernatant of the two mixture (at pH 7 and 8) was carried out using HPLC. The equal amount of matter of VA and CZ lost at pH 7 (12.05 and 11.51 mmol L^−1^) is in favour of a 1:1 molar interaction between the two antibiotics, as is the identical amount (in µmol) of the two antibiotics found in an analysis of a precipitate. This information is also coherent with the findings of Raju et al. [32] who also analysed the precipitate formed when mixing a 10 mg/mL solution VA with a 20 mg/mL solution of CZ by HPLC and using microbiological activity assay. They found the presence of both compounds in the precipitate, which also exhibited an antibiotic effect against both *S. aureus* and *E. coli* strains. The combination of all these results seemed to confirm that CZ and VA form an equimolar association which will precipitate before pH 7.3. Between 7.3 and 8.4, it would be the molecular part of the VA that would be the main source of the precipitation, as well as perhaps a marginal amount of VA/CZ precipitate formed by the interaction of residual positively charged VA with CZ.

The use of CD as carrier molecules for enhancing the solubility of drugs is now a well-known process, and the interaction can be characterized by various solubility diagram studies and analytical methods such as FT-IR, DSC, X-ray and NMR [36,37,38,39,40]. As Misiuk reported [22], it is possible to characterize the inclusion of CZ in a cage molecule using NMR by superimposing the spectrum of the free molecule with the spectrum of the molecule mixed with its cage. Our NMR data showed offsets as well as loss of resolution. This resulted in a broadening of the peaks of the spectrum of antibiotics mixed with their HPCD compared to the spectra of antibiotics alone, which is explained by a change in the environment of the molecule’s protons. From a theoretical point of view, this could have been due to different pHs between solutions, a difference in ionic strength, the viscosity of the medium, or the interaction with another molecule. Here, the pHs were strictly the same and the impact of viscosity was eliminated by diluting the solutions with a suitable buffer which did not change the trend of the results. In order to decide between a difference in ionic strength or an inclusion, a 2D analysis was necessary. It is possible to choose to verify the proximity of the protons of the molecule with those of the cage molecule via a NOESY such as Misiuk [22] or Ja’far et al. [41]. Here, DOSY (2D) [42,43,44], was preferred as described by Venuti et al. [45] in order to measure the difference in diffusivity between single molecules and the complex. Thus, by superimposing the water signals on the different spectra, the viscosity factor was eliminated. The shift in the signals of the spectra (lower chemical shift for the antibiotic with its HPCD) is the result of the increase in the apparent size, and therefore in the average molar mass, thus reflecting an interaction between the HPCDs and their corresponding antibiotics forming a larger complex. It is important to note in the case of the VA that its apparent size and therefore its average molar mass is greater than the apparent size of the HPβCD in the mixture. This is explained firstly because their molar mass is rather close but also secondly because the HPβCDs are in very large excess, meaning that in the solution there will be a great number of molecules that will not be able to interact with VA; however, all VA interacts with HPβCDs. As such, the average molar mass will be much less impacted in the case of HPβCDs, because of their large excess. The inclusion of VA and CZ in respectively βCD and γCD has already been studied by previous authors [20,21,22], who proved the reality of the complexation, using complementary methods. The NMR data provided in this study confirms their findings. Indeed, NMR is one of the most used and useful method to obtain reliable information on molecular interactions, as it allows the determination of specific signals of the host molecule, the included molecule and the complex. In addition, the combined use of 1H shifts analysis and DOSY experiments brings double confirmation of the inclusion [46]. It was not possible using only the data available from the DOSY analysis to suggest possible molecular structures of inclusion complexes. Because of the size of the complexes, this analysis would need to be performed using 2D ROESY [43]. Therefore, and also because this information did not pertain to the objective of the study (it would not help in resolving the interaction), this analysis was not performed.

After having characterized the inclusion of the antibiotics in HPCDs, we evaluated the impact of the concentration of HPβCD on the solubility profile of a 50 mg mL^−1^ VA solution at pH 8, in order to define the molar ratio (MR) of HPCD necessary to solubilize a high concentration of VA. We performed this phase solubility study to characterize the inclusion only for VA because it was the antibiotic that presented a visual precipitation when the pH was between 7.5 and 8.4. We choose to perform the phase solubility study with the data presented as in Figure 5 rather than as a Job’s plot as the precipitation of VA in presence of CDs seemed to be influenced by time and could occur after 15 min to 24 h. For CZ, as this molecule is already soluble for pH ranging between 3 and 9, a phase solubility study could not be performed. The results showed that the interaction phenomenon between VA and HPβCD is a dynamic phenomenon that does not lead to covalent bonding and that the phenomenon is reversible at a given pH. Indeed, the fact that the precipitation takes places (for lower MR) continuously after preparation, as shown in Figure 4 by the decrease of VA concentrations in solution over time, reflects an equilibrium inclined towards the dissociation of the HPβCD/VA complex. This may be due to the release of a VA molecule, which, by precipitating, will alter the balance and cause the release of other molecules. By saturating the medium with HPβCD, a shift of this balance in the direction of the formation of the complex seems to stabilise the inclusion. In our experimental conditions the molecular ratio (MR) required to solubilize all of the VA and therefore create a mixture without the formation of a precipitate during the time of the study was the ratio 10:1 (HPβCD:VA).

In order to study the average impact of various factors, including cyclodextrin concentrations, on the solubilisation of ATB mixtures, a reduced experimental design was set up. Initially, the results obtained via the preliminary and characterisation studies directed research towards a 10:1 MR (HPβCD:VA) in order to subsequently introduce a MR of HPγCD:CZ. Three main response factors were selected. Turbidity, which was the main discriminatory test, reflects the presence or absence of particles in suspension and therefore provides quantifiable information on the presence of precipitate. Thus, the target turbidity was for it to be as close to zero as possible. The size and number of subvisible particles was also measured and to be acceptable also has to be as small as possible since it will represent the number of particles in suspension in the solutions and therefore the number of insolubilized molecules. Lastly, osmolality was also measured, as this parameter should be, if possible, as close as possible to the value that can be found in contact with the eye, i.e., around 293 (iso-osmolar), knowing that a fairly wide tolerance exists. The use of an experimental design made it possible to greatly limit the number of experiments to be carried out while estimating the average impacts of our various parameters on the measured responses: only 84 experiments were carried out of 3888 possible combinations, which represents an economy of time and reduced reagents consumption. The results allowed the selection of two formulas to choose from. To choose between these formulations, several aspects were taken into account. First, the concentration corresponding to the HPβCD: VA at 5:1 MR (125 mg mL^−1^ of HPβCD) was the maximum limit of concentration proposed by the European Medicines Agency, under which there was no described ocular toxicity and is therefore considered safe [47,48]. Thus, concentrations greater than 125 mg mL^−1^ of HPβCD were above this limit. The use of higher concentrations would require a toxicity study which would have been long but above all irrelevant immediately since an alternative was possible with the addition of HPγCD at the MR of 3:1 (HPγCD: CZ). Additionally, the 10:1 MRs had higher osmolalities in comparison with the experiments containing both an HPβCD:VA 5:1 MR and an HPγCD: CZ 3:1 MR. This could have been a problem for future clinical applications, but as the eye does possess a high tolerance to the administration of solutions with a wide range of osmolalities [49,50], the United States Pharmacopoeia even giving a precise range of 171 to 1711 mOsm. kg^−1^ for adequate ocular tolerance [51], this should not be an issue. Finally, the solutions containing only HPβCD at a MR HPβCD: VA of 10: 1 at pH 8 were not stable for 24 h at 5 °C since a precipitation appeared after one or two hours, unlike the optimal formulation containing the two cyclodextrins, whose mixture remained physically stable for at least 48 h.

In order to decide between pH 7.5 and 8 for the final formulation, several elements were taken into account. A pH of 7.5 would be of greater interest because the medium is less alkaline, and therefore, closer to the optimum chemical stability pH of VA and especially of CZ [52,53]. However, the formulation at pH 8 was found to be physically stable at 5 °C for at least 48 h, and this was not the case for the same formulation at pH 7.5, which is closer to the incompatibility pH limit when using HPCD. This solubility limit at 5 °C could subsequently cause storage problems since VA and especially CZ are known for being chemically instable at ambient temperatures [54,55,56]. In addition, pH 7.5 is the lowest pH that could be obtained using this formulation method for which the solubilization of CZ and VA was possible. This could be explained by the affinity of species with each other. Indeed, the net charge of VA is +1 below pH 7.3 and the net charge of CZ is -1 below the same pH. If the affinity of this form of VA is higher for CZ than for HPCD, it would preferentially associate with CZ and precipitate instead of being complexed by the HPβCD. In addition, it has been shown that an interaction is possible between the contents and the container for some LDPE packaging articles sterilized with gamma rays, leading to acidification of the eye drops [57]; in our case, this acidification could lead us below our pH limit and therefore lead to precipitation if the buffering capacity is exceeded.

After defining the optimal conditions, a scale-up was carried out, and the results of pH, osmolality, turbidity measurements as well as subvisible particle counting confirmed the predictions of the DOE. It is, thus, possible to observe that these conditions give the best possible combination of results in comparison with the 84 other experiments. In addition, the solution resulting from the scale-up did not show any precipitation over a period of 48 h of refrigerated storage.

To verify that the formulation adopted did not show any loss of activity, bacteriological tests on the strains most found in the context of KB (*Staphylococcus aureus* and *Pseudomonas aeruginosa* [1,2,5,6]) were carried out. The antibacterial activity was also tested against *Escherichia coli* which has also been reported in bacterial keratitis and other ocular infections [58,59]. The three strains are also recommended as quality control by the French Society of Microbiology (SFM) and the EUCAST (European Committee of Antibiotic Susceptibility Testing) [60,61]. First, it is important to note that the concentrations are slightly below the expected concentrations of 25 mg/mL (between 21.64 mg/mL and 24.16 mg/mL) which is surely linked to the uncertainty of the measurement of volumes in the syringe as well as volume expansion. In addition, before and after 0.22 µm filtration the ATB concentration does not significantly decrease, which could have been the case if the HPCD/ATB complexes had been retained by the sterilizing filter. Bacteriological tests showed unchanged activity whether it was the antibiotic alone, the antibiotic in his HPCD or even the antibiotic in the final mixture. In addition, a synergistic effect of the two antibiotics as a mixture was possibly observed against the strains of *S. aureus* with larger diameters on average than those of the ATBs separately. This is faithful to what can be found in the literature [5,6] since the dual CZ/VA therapy makes it possible to target both the strains responsible for KB: Staphylococcus aureus (effect of CZ and VA) and Pseudomonas aeruginosa (effect of CZ).

In this study, we managed to develop an ophthalmic formulation combining two normally incompatible antibiotics in order to treat BK. The association of two antibiotics in one formula could reduce by half the number of administrations, thus facilitating its administration by nurses and improving compliance to the treatment by patients. We used phosphate buffer at a final concentration of 50 mM, in order to maintain pH and to limit its variation. This buffer was chosen to avoid potential incompatibilities using carbonate buffer with ceftazidime [13], and is commonly used in many preparations as well as commercialized eye-drops at a concentration varying from 1.3 to 111.2 mM [50,62]. HPβCD were used at their maximum allowed concentration, as described by the European Medicines Agency which is of 12.5% [47]. If higher concentrations of antibiotics (such as 50 mg mL^−1^) were needed, it is very likely that higher concentrations of CDs would be needed, which is not necessarily possible either from an ocular tolerance point of view, but also from a strictly chemical view as the limits of solubilities of the CDs could be reached. In addition, the use of HPγCD could be further investigated in terms of safety for ocular use even if the EMA does not prohibit its use in ocular formulation and it has been reported to be used or tested in many extemporaneous preparations [63] such as for example but not exhaustively Nepafenac [64], Dexamethasone [65] and amphotericin B eye drops [66]. It is also to note that the cost of the eye drops could unfortunately be higher than the typical eye drops, but this should be compared to the direct and indirect cost of the classical formulation. Irritancy tests have not yet been performed as they were not part of the objectives of this study, which aimed to investigate the potential use of cyclodextrins for blocking the problematic incompatibility between VA and CZ. As the formulation achieved this first goal, the next step would be to evaluate its stability using physical, chemical and biological analytical methods over time after storage at various temperatures before evaluating its potential effect on eye tissues. Concerning permeation tests, as the antibiotics are not destined to be absorbed by the eye tissues but to have an effect on the bacteria forming the surface infection, these tests are not yet of high priority, but could be performed in a pre-clinical phase.

## 4. Materials and Methods

### 4.1. Reactive and Reagents

Preparation of the test solutions: vancomycin chlorhydrate and ceftazidime pentahydrate powders were obtained from Vancomycin Mylan^®^ and Ceftazidime Mylan^®^ powder for injectable solution vials (Mylan, Cournon-d’Auvergne, France). Deionized water (Versylene^®^) was purchased from Fresenius Kabi (Louviers, France). HP-β-CD (CAS 128446-35-5) and HP-γ-CD (CAS 128446-34-4) were obtained from Sigma-Aldrich (St. Louis, MO, USA). Sodium dihydrogenophosphate dihydrate (NaH_2_PO_4_) (Batch 190298040, exp. 30 November 2021), and disodic monohydrogenophosphate dodecahydrate (Na_2_HPO_4_) (Batch 18129611, exp. 30 April 2023) were provided by Inresa (Bartenheim, France). Phosphoric acid at 850 mg mL^−1^ (H_3_PO_4_), and sodium hydroxide at 320 mg mL^−1^ (NaOH) (CAS: 1310-73-2), were purchased from Honeywell (Germany). Finally, deuterated water (D_2_O) and Tetra Deuteriated TrimethylSilylPropionate (TSPd_4_) were purchased from Eurisotop (St. Aubin, France).

### 4.2. Study Design

Firstly, a preliminary investigation of the CZ-VA incompatibility was realized. The potential impact of cyclodextrins in resolving incompatibilities between CZ and VA antibiotics was then studied by characterizing the inclusion of each antibiotic with its proposed corresponding HPCD by liquid NMR and complementary VA solubility test. After that, a design of experiments (DOE) was then performed to determine the impact of multiple parameters in order to inhibit the incompatibility and to define the best experimental condition to obtain a physically stable solution of the antibiotic mixture. Finally, the best solution given by the DOE was tested in a bacterial culture to verify the efficiency of the mixture compared to the individual solutions of antibiotics.

#### 4.2.1. Preparation of Solutions

##### Antibiotics Solutions

For all experiments, VA and CZ powders were reconstituted in deionized water in order to obtain a 200 mg m^−1^ solution. Those solutions were then diluted to 25 mg mL^−1^ (for the preliminary study) or 50 mg mL^−1^ (for the NMR analyses, VA precipitation study, DOE experiments and microbial activity measurement). These final dilutions were performed either in water, in 50 mM phosphate buffer aqueous solution or in HP-CD and phosphate buffer solution. The VA and CZ solution of 50 mg mL^−1^ were mixed in the final step at equal volumes to obtain a VA/CZ mixture at 25 mg mL^−1^ each. All adjustment to the desired pH was realized using a few microliters of NaOH or H_3_PO_4_ solutions with a SevenMulti™ pH-meter with an InLabTM Micro Pro glass electrode (Mettler-Toledo, Viroflay, France).

##### Phosphate Buffer Solution

Solutions of about 50 mM phosphate buffer were prepared in order to maintain the pH of the antibiotic solutions at the desired values. Phosphate solutions at pH 3; 4; 6 and 8 were prepared following the information given in Table 4 and completed to 50 mL with deionized water. Adjustment to the desired value was realized using few microlitres of NaOH or H_3_PO_4_ solution.

##### Hydroxypropyl Cyclodextrins in Buffered Aqueous Solution

HP-CD solution (γ and β) were prepared using the phosphate buffer solutions described previously. For each cyclodextrin, the appropriate quantity was weighed in order to obtain the proper molecular ratio for the corresponding antibiotic (HPγCD with CZ and VA with HPβCD), and dissolved in HPCD phosphate buffer solution to obtain a final volume of 50 mL. The concentration required to obtain a ratio of 1:1 with the antibiotic is respectively of 50.36 mg mL^−1^ and 144.5 mg mL^−1^ for respectively HPβCD and HPγCD.

#### 4.2.2. Preliminary Investigation of the Ceftazidime-Vancomycin Incompatibility

To investigate the nature of the incompatibility, pH variations ranging from 3 to 9 and 4 to 9 were applied to respectively VA and CZ 25 mg mL^−1^ solutions, as well as to a CZ/VA mixture. A visual examination was realized for each solution during the pH variation in front of a white and black panel.

To further determine the nature of precipitant of the antibiotic mixture, a quantification of CZ and VA was performed in the solution supernatant of a CZ/VA mixture in phosphate buffer solution at 50 mM adjusted to pH 7 and 8 by high performance liquid chromatography (HPLC). In order to give additional proof about the composition of the precipitate, some of it was also recovered (separated from the supernatant by centrifugation) and dissolved with a pH 9 buffer solution. The resulting solution was also analysed by HPLC.

The system used was a Prominence-I LC2030C 3D with diode array detection (Shimadzu France SAS, Marne La Vallée, France) and the associated software used to record and analyse the chromatograms was LabSolutions^®^ version 5.82. After precipitation, each solution was centrifuged at 5000 rpm for 10 min, then diluted 1/10th in deionized water. The separation column used was a Nucleodur SB Gravity C18 (250 × 4.6 mm, 5 µm) column (Macherey Nagel, Hoerdt, France) and associated guard column (12.5 × 4.6 mm). The mobile phase was an acetonitrile/ammonium acetate 100 mM aqueous buffer adjusted with acetic acid to pH 5.8 (10/90% *v*/*v*) in which the acetonitrile was HPLC quality (Chromasolv^®^ for HPLC; Honeywell^®^, Roissy CDG, France) and the water was sterile deionized (Versylene^®^; Fresenius Kabi France, Louviers, France). The flow rate through the column for the analysis was set at 1.2 mL/min, with the column thermo-regulated to a temperature of 25 °C. The injection volume was 20 μL. The quantification wavelength was set up at 220 nm for vancomycin and 256 nm for ceftazidime. The method used allows vancomycin quantification with a mean accuracy of 99.04 ± 4.5%, repeatability’s relative standard deviation (RSD) of 0.94% and intermediate precision’s RSD of 1.07%. As for ceftazidime quantification, the method’s mean accuracy was of 99.04 ± 2.8%, repeatability’s relative standard deviation (RSD) of 1.2% and intermediate precision is RSD of 2.61%. It allows its quantification from 50 to 250 μg mL^−1^ with a determination coefficient R^2^ higher to 0.998 for both antibiotics. Method validation data are provided in the Supplementary Materials Files S1 and S2 (raw data).

#### 4.2.3. Antibiotics Inclusion Characterization in HP-CD

##### NMR Analyses

For each antibiotic, three kinds of solution and their 10 fold dilution in deuterated phosphate buffer solution were prepared: 1 mL of 200 mg mL^−1^ VA solution with 3 mL pH 3 buffer solution; 1 mL water with 3 mL HPβCD in pH 3 buffer solution, 1 mL VA with 3 mL HPβCD in pH 3 buffer solution; 1 mL CZ with 3mL pH 4 buffer solution, 1mL water with 3mL HPγCD in pH 3 buffer solution; and 1 mL CZ with 3 mL HPγCD in pH 4 buffer solution.

##### ^1^H NMR-Spectroscopy Chemical Shifts Measurements

The 1H NMR spectra were recorded at 298K on a Bruker AVANCE III HD 500 MHz spectrometer equipped with Bruker 5 mm inverse probe TXI (^1^H/^13^C/^15^N) with z-gradient coil probe. Solution of TSPd4 in D2O was used as internal reference for chemical shifts. To eliminate possible interactions between TSPd4 and the host molecule, the internal reference was introduced into a coaxial insert itself placed in the NMR tube. For all samples, a one dimensional ^1^H NMR spectrum was acquired using a ZGPRESAT sequence with water pre-saturation at low power. A total of 8 or 16 scans were collected with a 90 °C impulsion time of 10.2 µs, a 30 s relaxation time, an acquisition of 4.09 s, a spectral window of 8000 Hz and 65 K data points zero-filled to 128 K before Fourier transformation with 0.3 Hz line broadening. In addition, the determination of the average molar mass of the two CDs was estimated by an assay with an internal reference (TSP-d4).

##### ^1^H NMR-Diffusion Measurements (^1^H-DOSY)

The DOSY spectra were recorded at 298,0K (MeOD calibration and an air flow control of 545 l.h^−1^) on a Bruker AVANCE III 500 MHz spectrometer with a Prodigy TCI ^1^H/^13^C/^15^N/D cryoprobe. DOSY experiments were performed using the bipolar longitudinal eddy current delay pulse sequence (ledbpgp2s) [42]. The durations of the magnetic field pulse gradients were optimized between 1 to 2 ms (to obtain complete dephasing of the signal with the maximum gradient strength) with 5 ms eddy current delay and spoil gradients of 600 µs with 17:13% ratio. The pulse gradient has been incremented from 5 to 95% (to 65% for H_2_O diffusion) of the maximum strength in a linear ramp. The diffusion times have been optimized between to 40ms (for H_2_O diffusion) to 100 ms (for antibiotics and cyclodextrins diffusion). Each DOSY experiment is a series of 16 spectra with 16K data points, 4 dummy scans, 8 to 32 scans and a relaxation delay of 5 s. After Fourier transformation, phasing and baseline correction, the diffusion dimension has processed with the Bruker Dynamics Center software (2.7.1 version, Bruker, Ettlingen, Germany). For each sample, the residual water signal diffusion coefficient was measured and set as reference of DOSY spectra and diffusivity calculations [43,44].

##### Influence of HPβCD on Inclusion of VA and Its Precipitation

As vancomycin precipitated between pH 7.3 to 8.4, the impact of HPβCD in the solubilisation of vancomycin was studied at pH 8. For this purpose, a bulk solution of 380 mM HP-β-CD in a 50 mM phosphate buffer solution adjusted to pH 8 was prepared from which various volumes were then added to 1 mL of a 200 mg/mL of VA and then completed with 50 mM phosphate buffer solution to obtain at the end 4 mL of a 50 mg/mL of VA, as presented in Table 5.

Once the final pH was adjusted to 8, each solution was stirred for 30 min and then centrifuged at 5000 rpm for 10 min, before being diluted in deionized water in order to quantify VA in the supernatant phase using a UV spectrophotometer (V670, Jasco France SAS, Lisses, France). The quantification was performed after 15 min, 24 and 48 h after preparation in which centrifugation was realized before each analyses.

The validated quantification method had a calibration curve of y = 0.00447968x − 0.0541038, where x is the absorbance at 280 nm and y is VA concentration, with a R^2^ of 0.999 and standard error of 1.58, allowing the quantification of VA between 75 and 175 µg mL^−1^.

#### 4.2.4. Determination of the Best Preparation Method by Design of Experiment

The impact of multiple parameters on VA/CZ precipitation was studied using a DOE method following a D-optimal design. Seven parameters were chosen to be evaluated in an experimental design: concentrations of HPβCD and of HPγCD, pH of the HPβCD/VA and HPγCD/CZ mixture, stirring duration of HPβCD/VA and HPγCD/CZ mixture and final pH of the antibiotic mixture. The relative impact of these seven parameters was tested on five responses: visual aspect of the preparation, turbidity 2 categories of subvisible particles (bigger and smaller than 10 µm) and osmolality. For visual aspect assessment, a semi-quantitative (subjective) score was given between 0 and 10 (with 0—no particles, 10—precipitation). Turbidity was measured using a 2100Q Portable Turbidimeter (Hach Lange, Marne La Vallée, France). Subvisible particles counting was performed using a HIAC Royco 9703 (Hach Lange, Noisy le Grand, France) equipped with a HRLD 400 EC detector, and osmolality was measured on 20 µL samples using a freezing point osmometer (Model 2020, Advanced instruments Inc., Radiometer, SAS, Neuilly Plaisance, France).

The details concerning each parameter are presented in Table 6. In total, 84 experiments were carried out (see Supplementary Materials S1). The calculations of each parameters impact were performed using Modde^®^ software (Sartorius, Goettingen, Germany), which was also used to establish the best preparation conditions based on turbidity values, visual examination and osmolality of the preparation.

#### 4.2.5. Validation of the Preparation Method

The two best set of experimental conditions, established by the DOE, were tested in order to prepare 48 mL of the CZ/VA mixture solution. For this purpose, 24 mL of each antibiotic solution (VA and CZ) were prepared and mixed together according to the conditions defined by the DOE. The sterilization of the final solution was realized by filtration through 0.22 µm filter in aseptic conditions, and the filtrated solution was quantified and compared to concentration before filtration. The filtrated solution was also placed in room temperature and at 5 °C to check for the absence of any precipitation after 24 h, assessed by visual examination and turbidity measurements.

#### 4.2.6. Verification of the Efficiency Mixture of the Optimized Formula

The efficiency of the best formula determined by DOE and after validation of the preparation method was verified by disk diffusion method against *E. coli* ATCC 25922, *P. aeruginosa* ATCC27853 and *S. aureus* ATCC29213. For this purpose, a sterile solution of CZ-VA mixture was prepared and then compared to 50 mM phosphate pH 8 buffer solution used as negative control, and to VA and CZ separate solutions used as reference response. A total of 1 µL of the tested solution was applied on a blank disk set on a Mueller-Hinton agar plate inoculated with a 0.5 McF bacterial suspension. The growth-inhibitory zone was measured after 18 h of incubation at 35 °C. Each test was performed in triplicate.

## 5. Conclusions

The data provided in this study supports the hypothesis that the usually incompatible mixture of VA and CZ is linked to the formation of an equimolar precipitate between the two oppositely charged drugs. The results of the DOE study showed that HPCD can resolve this incompatibly when the pH is higher than 7.5, and that an ophthalmic formulation combining these two essential antibiotics with HPCD can be made. It is has been proven that the antibiotics included in the HPCD maintained the bacterial activities of both molecules, which is essential to treat BK. It would, however, be essential to demonstrate the safety of this new formulation and its stability in order to be able to consider its use for human medicine.

## Figures and Tables

**Table 1:** Ceftazidime (CZ) and vancomycin (VA) concentrations in the supernatant after centrifugation of the solution mixture, as a function of the mixture pH. RSD: relative standard deviation.

	Initial Concentration (mg mL^−1^)	pH of the VA/CZ Mixture	Concentration (mg mL^−1^) in the Supernatant (*n* = 3)		% of Initial Concentration	Initial Concentration (mmol L^−1^)	Loss (mmol L^−1^)
			Average	RSD			
Vancomycin	24.16	7	6.69	6.26%	27.7%	16.67	12.05
		8	12.08	6.92%	50.0%		8.33
Ceftazidime	21.95	7	15.66	6.14%	71.4%	40.16	11.51
		8	20.69	6.62%	94.3%		2.31

**Table 2:** Parameters relative impact on the studied responses according to the design of experiments. Hydroxypropyl-β-cyclodextrins: HPβCD; hydroxypropyl-γ-cyclodextrins: HPγCD; VA: vancomycin; CZ: ceftazidime.

Parameters	Average Response (When Parameters Vary from Minimum to Maximum)				
	Visual Examination	Turbidity	≥10 µm Particles Count	≤10 µm Particles Count	Osmolality
HPβCD Concentration	−0.39	−50.03	−140	7	103
HPγCD Concentration	−0.47	−56.09	−787	−235	73
pH mixture of HPβCD/VA	0.11	22.76	498	417	−8
pH mixture of HPγCD/CZ	0.35	33.14	109	176	−41
Stirring duration of HPβCD/VA	−0.28	−44.02	157	345	14
Stirring duration of HPγCD/CZ	0.3	49.08	−219	36	−2
final pH of the mixture	−2.33	−389.86	−581	−261	14

**Table 3:** Inhibition diameters of the tested solutions against *Escherichia coli*, *Staphylococcus aureus* and *Pseudomonas aeruginosa* cultures. Mean ± standard deviation, *n* = 3. Hydroxypropyl-β-cyclodextrins: HPβCD; hydroxypropyl-γ-cyclodextrins: HPγCD; VA: vancomycin; CZ: ceftazidime.

	Inhibition Diameters (mm)		
	*Staphylococcus aureus*	*Escherichia* *coli*	*Pseudomonas* *aeruginosa*
HPγCD	6 ± 0	6 ± 0	6 ± 0
HPβCD	6 ± 0	6 ± 0	6 ± 0
CZ	18 ± 1	27 ± 1	26 ± 1
VA	17 ± 1	6 ± 0	6 ± 0
CZ/HPγCD	17 ± 1	27 ± 1	25 ± 1
VA/HPβCD	16 ± 1	6 ± 0	6 ± 0
CZ/HPγCD + VA/HPβCD mixture (formula B)	21 ± 1	27 ± 0	26 ± 0

**Table 4:** Phosphate buffer solution composition in 50 mL at various pH.

Phosphate Buffer Solution	pH 3	pH 4	pH 6	pH 8
H_3_PO_4_ at 85 mg mL^−1^ (µL)	2000	212	-	-
NaH_2_PO_4_, 2H_2_O (mg)	205	230	202.5	1.1
Na_2_HPO_4_, 12H_2_O (mg)	-	-	73.7	505

**Table 5:** Preparation of vancomycin (VA)/2-hydroxypropyl-β-cyclodextrin (HPβCD) solution for the study of the impact of HPβCD on precipitation.

Volume of 380 mM HPβCD solution at pH 8 (mL)	0	0.819	1.364	1.910	2.182	2.455	2.728	3.000
Volume 50 mM phosphate buffer solution pH 8 (mL)	3.000	2.181	1.636	1.090	0.818	0.545	0.272	0
Volume of VA 200 mg/mL solution (mL)	1	1	1	1	1	1	1	1
Corresponding ratio HPβCD:VA	0:1	3:1	5:1	7:1	8:1	9:1	10:1	11:1

**Table 6:** Parameters studied for their impact on the precipitation of ceftazidime (CZ)/vancomycin (VA) mixture solution. HPβCD: 2-hydroxypropyl-β-cyclodextrin; HPγCD: 2-hydroxypropyl-γ- cyclodextrin.

Parameters	Type	Studied Levels
HPβCD ratio (compared to VA)	Multilevel	0; 2; 5 and 10
HPγCD ratio (compared to CZ)	Multilevel	0; 1 and 3
VA/HPβCD pH mixture	Quantitative	3 to 6
CZ/HPγCD pH mixture	Quantitative	4 to 8
VA/HPβCD mixture time (hours)	Multilevel	0.5; 1 and 2
CZ/HPγCD mixture time (hours)	Multilevel	0.5; 1 and 2
pH of the final solution	Multilevel	7; 7.5; 8 and 8.5

## Data Availability

All raw data is provided in the Supplementary Data File S2.

## Supplementary Materials

The following are available online at https://www.mdpi.com/article/10.3390/ijms221910538/s1: Supplementary Data File S1 (containing the additional data described in the manuscript) and S2 (containing all raw data).
