# Supplementary material for: Cyclodextrins Allow the Combination of Incompatible Vancomycin and Ceftazidime into an Ophthalmic Formulation for the Treatment of Bacterial Keratitis
Source: Int J Mol Sci. 2021 Sep 29;22(19):10538. doi: 10.3390/ijms221910538 (PMC8508691; doi:10.3390/ijms221910538)
Supplement: Supplementary file 1 [file ijms-22-10538-s001.zip › Supplementary materials_Rev3/Supplementary materials S1.pdf]

Cyclodextrins allow the combination of incompatible vancomycin and ceftazidime into an ophthalmic formulation for the treatment of bacterial keratitis

Yassine Bouattour[1], Florent Neflot-Bissuel[2], Mounir Traïkia [3], Anne-Sophie Biesse-Martin[3], Robin Frederic[4], Mouloud Yessaad[2], Mireille Jouannet[2], Mathieu Wasiak[2], Philip Chennell[1*] and Valerie Sautou[1]

[1] Université Clermont Auvergne, CHU Clermont Ferrand, Clermont Auvergne INP, CNRS, ICCF, F-63000 Clermont-Ferrand, France; ybouattour@chu-clermontferrand.fr; pchennell@chu-clermontferrand.fr; vsautou@chu-clermontferrand.fr

[2] CHU Clermont-Ferrand, Pôle Pharmacie, F-63000 Clermont-Ferrand, France; bissuelflorent@orange.fr; myessaad@chu-clermontferrand.fr; mjouannet@chu-clermontferrand.fr; mwasiak@chu-clermontferrand.fr

[3] Université Clermont Auvergne, CNRS, SIGMA-Clermont, ICCF, F-63000 Clermont-Ferrand, France. mounir.traikia@uca.fr; a-sophie.biesse-martin@uca.fr

[4] Université Clermont Auvergne, Inserm U1071, INRA USC2018, Clermont-Ferrand, France; frobin@chu-clermontferrand.fr

* Correspondence: pchennell@chu-clermontferrand.fr

1. Illustration of the pH dependant nature of the precipitation between ceftazidime and vancomycin

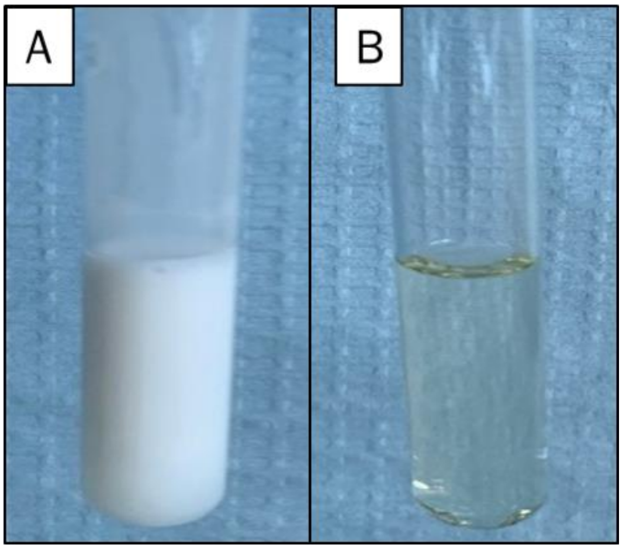

Figure 1. Mixture of 25 mg mL-1 solution of ceftazidime and vancomycin at A) pH below 8.4 and B) pH higher than 8.4

2. Validation of ceftazidime and vancomycin quantification method

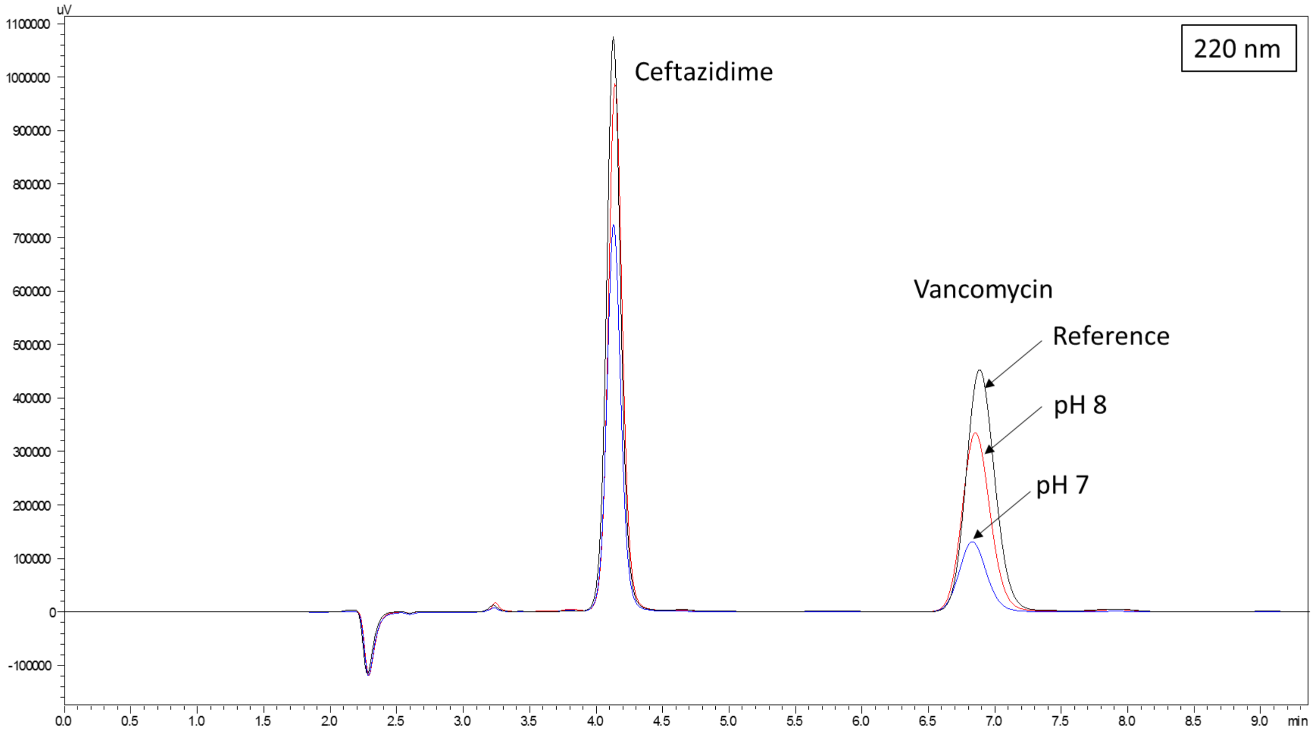

Figure 2: Chromatograms at 220 nm wavelength detection of mixture of ceftazidime and vancomycine at pH 7 and pH 8

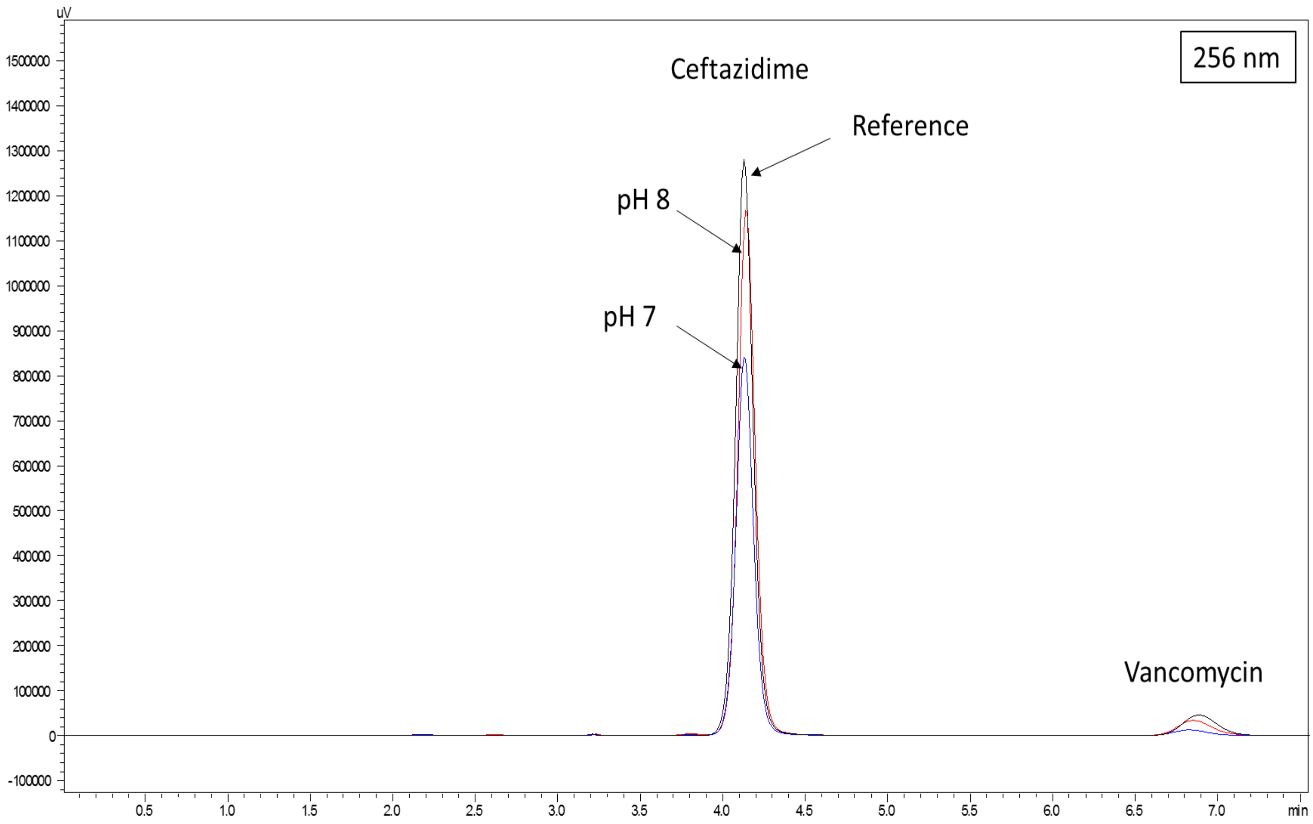

Figure 3 Chromatograms at 256 nm wavelength detection of mixture of ceftazidime and vancomycin at pH 7 and pH 8

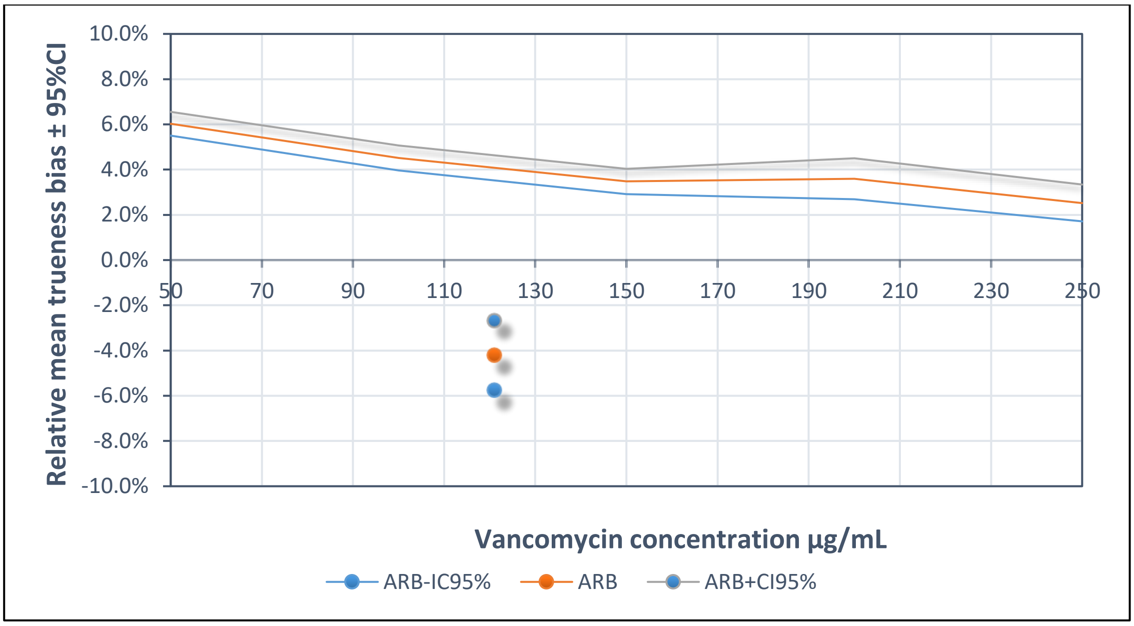

Figure 4 : Accuracy profile of Vancomycin validation. ARB: Average relative trueness bias. CI: confidence interval

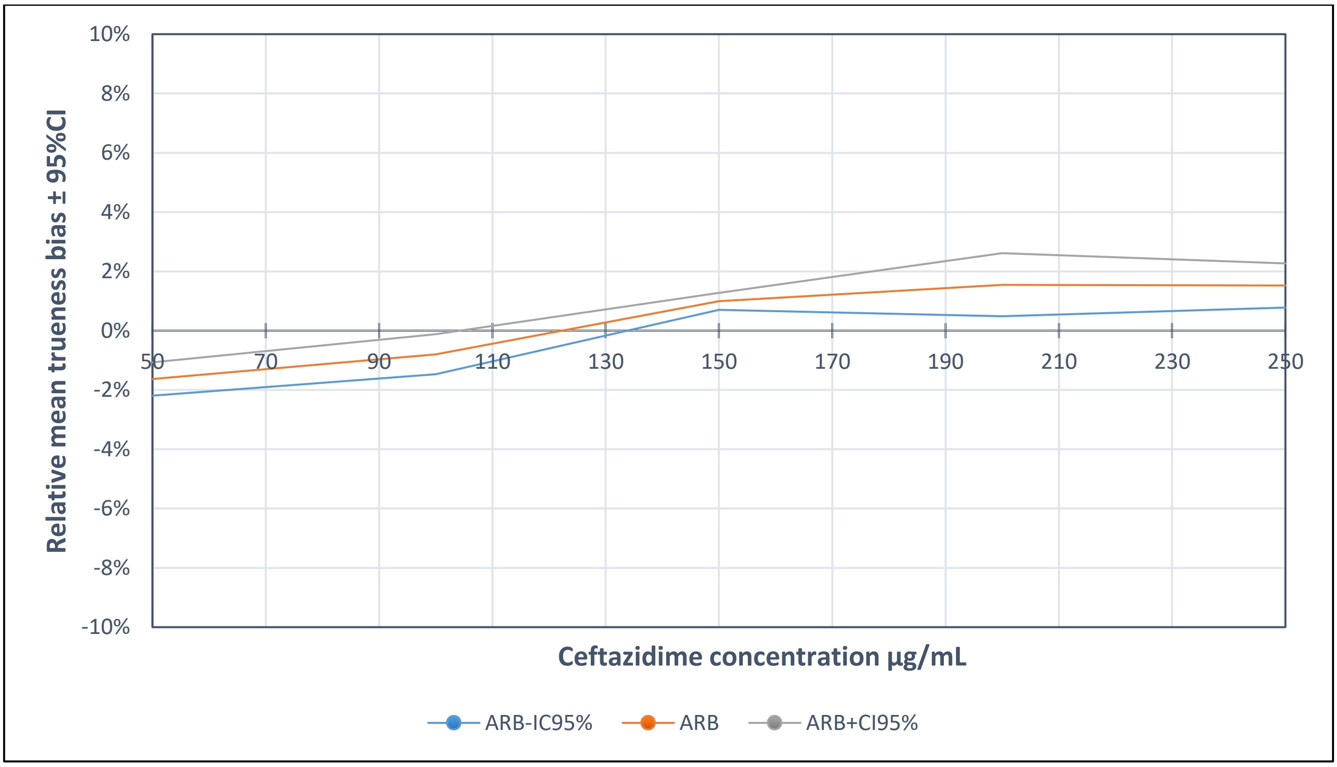

Figure 5 Accuracy profile of Ceftazidime validation. ARB: Average relative trueness bias. CI: confidence interval

3. Design of experiment

*Table 1: Experimental design realized to calculate the impact of studied parameters on turbidity, particles count and osmolality. NA: Not applicable ; * : measurement error*

	Parameters							Results					
**Exp No**	**HPβCD ratio (compared to VA)**	**HPγCD ratio (compared to CZ)**	**VA/ HPβCD pH mixture**	**CZ/ HPγCD pH mixture**	**VA/ HPβCD mixture time**	**CZ/ HPγCD mixture time**	**pH of the final solution**	**Visual examination**	**Turbidity (FNU)**	**Particules < 10 µm**	**particule > 10 µm**	**Osmolality (mOsmol/kg)**	**Final pH**
1	0	0	6	4	0,5	0,5	7	9	1000	NA	NA	211	7
2	5	0	3	4	0,5	0,5	7	9	1000	NA	NA	366	7
3	0	3	4	8	0,5	0,5	7	9	1000	NA	NA	315	7
4	5	3	6	4	0,5	0,5	7	9	1000	NA	NA	633	7
5	5	0	6	8	2	0,5	7	9	1000	NA	NA	265	7
6	0	3	3	4	2	0,5	7	9	1000	NA	NA	399	7
7	5	3	3	8	2	0,5	7,5	9	1000	NA	NA	518	7,5
8	5	0	6	8	0,5	2	7	9	1000	NA	NA	265	7
9	0	3	6	6	0,5	2	7	9	1000	NA	NA	327	7
10	5	3	3	4	0,5	2	7	8	1000	NA	NA	684	7
11	0	0	6	8	2	2	7	9	1000	NA	NA	154	7
12	5	0	6	4	2	2	7	9	1000	NA	NA	329	7
13	5	0	3	8	2	2	7	9	1000	NA	NA	269	7
14	5	3	6	8	2	2	7,5	9	1000	NA	NA	558	7,5
15	0	0	3	8	1	2	7	9	1000	NA	NA	150	7
16	5	3	4	6	2	0,5	7,5	3	7,58	4521	953,16	563	7,5
17	2	3	3	8	0,5	2	7,5	9	1000	NA	NA	414	7,5
18	0	1	4	4	1	2	7,5	9	1000	NA	NA	277	7,5
19	0	1	3	8	0,5	1	7,5	9	1000	NA	NA	201	7,5
20	5	0	6	8	0,5	0,5	8,5	3	7,16	6421,33	790,01	300	8,5
21	0	3	3	4	0,5	0,5	8,5	3	6,92	2708,16	673,34	494	8,5
22	5	3	3	8	0,5	0,5	8,5	2	4,57	2509,16	614,67	575	8,5

23	0	0	6	4	2	0,5	8,5	3	7,21	7425,83	1729,17	281	8,5
24	0	0	3	8	2	0,5	8,5	3	4,22	5731,33	865,01	185	8,5
25	5	0	3	4	2	0,5	8,5	3	6,88	3740,5	494,66	403	8,5
26	5	3	4	4	2	0,5	7,5	4	17	6097	863,32	598	7,5
27	0	0	6	8	0,5	2	8,5	3	6,96	3160,33	655,33	182	8,5
28	5	0	6	4	0,5	2	8,5	3	3,09	2804,67	662,84	368	8,5
29	5	0	3	8	0,5	2	8,5	3	3,04	2592,84	358,33	288	8,5
30	5	3	6	8	0,5	2	7,5	9	1000	NA	NA	525	7,5
31	0	0	3	4	2	2	8,5	3	7	3662,16	933,49	260	8,5
32	5	0	6	8	2	2	8,5	3	4,6	2194,33	607,34	291	8,5
33	0	3	6	4	2	2	8,5	3	5,8	2765	604,16	476	8,5
34	0	3	3	8	2	2	8,5	3	6,4	5497,16	1065,66	344	8,5
35	5	3	3	4	2	2	8,5	2	5,88	3022	875,16	693	8,5
36	2	3	6	6	2	2	7,5	3	8,74	9006,17	1630,66	656	7,5
37	0	3	6	8	0,5	0,5	8	3	4,42	3220	665,66	349	8
38	5	3	6	4	0,5	0,5	7,5	9	1000	NA	NA	506	7,5
39	5	3	6	4	2	0,5	7,5	3	7,82	5108,67	5247,83	639	7,5
40	5	3	6	4	0,5	2	7,5	3	9,2	7418,67	513,84	610	7,5
41	5	1	6	4	2	2	7,5	9	1000	NA	NA	439	7,5
42	5	1	6	4	2	0,5	7,5	9	1000	NA	NA	440	7,5
43	2	3	6	4	2	0,5	7,5	7	1000	NA	NA	490	7,5
44	2	3	3	4	2	2	7,5	5	512	NA	NA	550	7,5
45	2	1	3	4	2	2	7,5	9	1000	NA	NA	365	7,5
46	2	1	3	4	0,5	0,5	7,5	9	1000	NA	NA	347	7,5
47	2	1	3	4	0,5	0,5	8	3	3,79	5388,5	833,32	382	8
48	2	1	6	6	2	2	8	3	3,93	4179,84	1256	311	8
49	2	1	3	8	2	2	8	3	6,23	5773	1338,33	289	8
50	5	3	3	6	0,5	2	8	3	2,05	1275,5	260,35	533	8
51	2	1	3	6	2	0,5	8	3	5,55	3332	561,84	390	8

52	2	1	4	8	2	2	8	3	4,05	4340,17	1179,17	284	8
53	2	1	6	8	2	2	8	3	3,33	2872,83	7650,01	285	8
54	2	1	4	4	0,5	0,5	8	3	3,8	3912	856,33	375	8
55	2	1	4	4	2	2	7,5	9	1000	NA	NA	330	7,5
56	2	3	6	4	2	2	7,5	3	4,2	3214,17	1043	473	7,5
57	0	0	6	4	2	2	8	9	1000	NA	NA	225	8
58	0	3	6	4	2	2	7,5	7	1000	NA	NA	*	7,5
59	0	3	6	4	2	2	8	9	1000	NA	NA	409	8
60	2	3	6	6	2	0,5	7,5	9	1000	NA	NA	429	7,5
61	5	1	6	8	2	2	7,5	9	1000	NA	NA	367	7,5
62	0	3	6	8	2	2	7,5	9	1000	NA	NA	300	7,5
63	5	3	3	4	2	0,5	7,5	3	3,5	2135,67	498,01	687	7,5
64	5	3	3	4	2	0,5	7,5	3	3,3	2056,66	740,84	673	7,5
65	5	3	3	4	2	0,5	7,5	3	3,5	2135,67	498,01	687	7,5
66	5	3	3	4	2	0,5	8	3	2,35	1572,5	146,32	760	8
67	3	3	4	6	2	2	7,5	9	1000	NA	NA	443	7,5
68	10	0	3	4	2	0,5	7	9	1000	NA	NA	*	7
69	10	0	6	4	2	0,5	7	9	1000	NA	NA	*	7
70	10	0	3	8	2	0,5	7	9	1000	NA	NA	*	7
71	10	0	6	8	2	0,5	7	9	1000	NA	NA	*	7
72	10	0	3	4	2	0,5	7,5	3	2,76	3704	478,75	458	7,5
73	10	0	6	4	2	0,5	7,5	9	1000	NA	NA	*	7,5
74	10	0	3	8	2	0,5	7,5	7	692	NA	NA	*	7,5
75	10	0	6	8	2	0,5	7,5	9	1000	NA	NA	*	7,5
76	10	0	3	4	2	0,5	8	3	2,62	1502	520,5	508	8
77	10	0	6	4	2	0,5	8	3	3,26	1607,75	444,5	751	8
78	10	0	3	8	2	0,5	8	3	3,83	2335,5	467,5	899	8
79	10	0	6	8	2	0,5	8	3	5,38	3607,75	558	962	8
80	5	1	4,5	6	1	1	8	3	2,25	3585,67	490,66	425	8

81	5	1	4,5	6	1	1	8	3	2,5	2578	419	441	8
82	5	1	4,5	6	1	1	8	3	2,7	4978,17	541,84	447	8
83	5	1	4,5	6	1	1	8	3	2	2430,83	512,5	426	8
84	5	1	4,5	6	1	1	8	3	2,54	4265,34	727,44	409	8

4. Nuclear magnetic resonance shift and chemical shifts analysis: peak assignments for ceftazidime and the inclusion complex of ceftazidime / Hydroxypropyl-γ-cyclodextrins

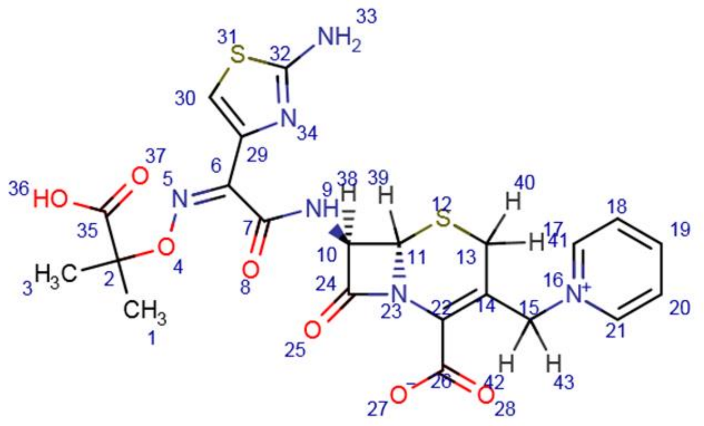

Figure 6 : Peak assignments for ceftazidime (^{1}H RMN)

	CZ		CZ + HPγCD		Chemical shifts	Attribution
Peak	ν(F1) [ppm]	Intensity [rel]	ν(F1) [ppm]	Intensity [rel]	(Δδ, ppm)	(^{1}H)
1	8.988	3.380	8.886	1.280	**-0.102**	**17, 21**
2	8.600	2.070	8.497	0.710	**-0.103**	**19**
3	8.119	3.110	8.016	1.230	**-0.103**	**18, 20**
4	7.027	11.800	6.890	1.580	**-0.137**	**30**
5	5.890	2.790	5.774	0.580	**-0.117**	**38**
6	5.600	2.040	5.489	0.450	**-0.111**	**43**
7	5.401	1.850	NA	NA	NA	**39**
8	5.320	4.200	NA	NA	NA	**42**
9	3.661	1.890	NA	NA	NA	**41**
10	3.268	2.100	3.146	0.420	**-0.123**	**40**
11	1.526	15.000	1.431	5.480	**-0.095**	**1,1,1 3,3,3**

Figure 2 : Chemical shifts and attribution for the protons of ceftazidime and ceftazidime / Hydroxypropyl-γ-cyclodextrins.

Table 2: Summary table of spectral shifts between Vancomycin spectrum without and with HPβCD. NA: Not applicable

Vancomycin			Vancomycin + HPβCD			Shifts
Peak	ν(F1) [ppm]	Intensity [rel]	Peak	ν(F1) [ppm]	Intensity	
1	7.7674	6.28	1	7.7105	0.04	**-0.057**
2	7.6643	4.06	2	7.6018	0.03	**-0.063**
3	7.6145	4.07	3	7.519	0.04	**-0.095**
4	7.5999	4.07	4	NA	NA	NA
5	7.3437	3.98	5	7.235	0.01	**-0.109**
6	7.1943	0.75	6	7.1045	0.02	**-0.090**

7	6.9456	0.98	7	6.866	0.01	**-0.080**
8	6.5957	0.98	8	6.4902	0.12	**-0.106**
9	6.5373	1.13	9	6.4316	0.11	**-0.106**
10	6.2562	2.18	10	6.1547	0.01	**-0.102**
11	5.6345	15	11	NA	NA	NA
12	5.5804	3.75	12	NA	NA	NA
13	5.479	1.54	13	NA	NA	NA
14	5.3933	1.42	14	NA	NA	NA
15	4.9455	0.86	15	NA	NA	NA
16	4,6325	2,24	16	NA	NA	NA
17	4,3166	1,02	17	NA	NA	NA
18	4,147	0,66	18	NA	NA	NA
19	3,9111	0,6	19	NA	NA	NA
20	3,8417	2,27	20	NA	NA	NA
21	3,725	3,09	21	NA	NA	NA
22	3,5119	1,75	22	NA	NA	NA
23	2,8213	0,74	23	2,7334	0,36	**-0,088**
24	2,1199	0,57	24	2,0165	0,06	**-0,103**
25	1,8349	2,94	25	1,7631	0,04	**-0,072**
26	1,7635	4,02	26	1,6485	0,03	**-0,115**
27	1,7537	0,67	27	NA	NA	NA
28	1,6298	0,91	28	NA	NA	NA
29	1,6225	0,75	29	NA	NA	NA
30	1,4932	1,41	30	1,4199	0,05	**-0,073**
31	1,2193	1,29	31	NA	NA	NA
32	1,2114	1,12	32	NA	NA	NA
34	0,9045	1,85	34	0,8192	0,32	**-0,085**
